# Understanding multiscale structure–property correlations in PVDF-HFP electrospun fiber membranes by SAXS and WAXS†

**DOI:** 10.1039/d1na00503k

**Published:** 2021-11-15

**Authors:** Anjani K. Maurya, Eloïse Mias, Jean Schoeller, Ines E. Collings, René M. Rossi, Alex Dommann, Antonia Neels

**Affiliations:** Empa, Swiss Federal Laboratories for Materials Science and Technology, Center for X-Ray Analytics Lerchenfeldstrasse 5 9014 St. Gallen Switzerland antonia.neels@empa.ch; Empa, Swiss Federal Laboratories for Materials Science and Technology, Laboratory for Biomimetic Membranes and Textiles Lerchenfeldstrasse 5 9014 St. Gallen Switzerland; ARTORG Center for Biomedical Engineering Research, University of Bern Murtenstrasse 50 3008 Bern Switzerland; ETH Zürich, Department of Health Science and Technology 8092 Zürich Switzerland; Department of Chemistry, University of Fribourg Avenue de l'Europe 20 1700 Fribourg Switzerland

## Abstract

Electrospinning is a versatile technique to produce nanofibrous membranes with applications in filtration, biosensing, biomedical and tissue engineering. The structural and therefore physical properties of electrospun fibers can be finely tuned by changing the electrospinning parameters. The large parameter window makes it challenging to optimize the properties of fibers for a specific application. Therefore, a fundamental understanding of the multiscale structure of fibers and its correlation with their macroscopic behaviors is required for the design and production of systems with dedicated applications. In this study, we demonstrate that the properties of poly(vinylidene fluoride-*co*-hexafluoro propylene) (PVDF-HFP) electrospun fibers can be tuned by changing the rotating drum speed used as a collector during electrospinning. Indeed, with the help of multiscale characterization techniques such as scanning electron microscopy (SEM), small-angle X-ray scattering (SAXS), and wide-angle X-ray scattering (WAXS), we observe that increasing the rotating drum speed not only aligns the fibers but also induces polymeric chain rearrangements at the molecular scale. Such changes result in enhanced mechanical properties and an increase of the piezoelectric β-phase of the PVDF-HFP fiber membranes. We detect nanostructural deformation behaviors when the aligned fibrous membrane is uniaxially stretched along the fiber alignment direction, while an increase in the alignment of the fibers is observed for randomly aligned samples. This was analyzed by performing *in situ* SAXS measurements coupled with uniaxial tensile loading of the fibrous membranes along the fiber alignment direction. The present study shows that fibrous membranes can be produced with varying degrees of fiber orientation, piezoelectric β-phase content, and mechanical properties by controlling the speed of the rotating drum collector during the fiber production. Such aligned fiber membranes have potential applications for neural or musculoskeletal tissue engineering.

## Abstract 2

Multiscale structure–property correlations in PVDF-HFP electrospun fiber membranes.

## Graphical abstract

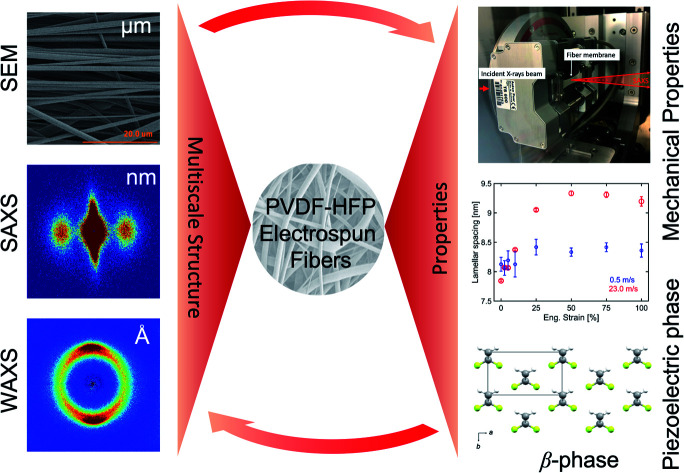

## Introduction

Electrospinning is a simple, and cost-effective method to produce fiber membranes for various applications such as filtration, drug delivery, biosensing, and tissue engineering due to the inherent advantages of a high surface-to-volume ratio and high porosity.^1–8^ Fiber membranes have gained ample attention of researchers in recent times as a scaffold for tissue engineering applications due to their abilities to mimic the extracellular matrix of natural tissues more accurately than macro- or micro-scale biomaterials.^9–12^ For example, aligned fiber membranes have shown promising response for neural and musculoskeletal tissue engineering as aligned fibers significantly induce neurite outgrowth and promote cell migration compared to randomly oriented fiber membranes.^13–19^ However, scaffolds for neural and musculoskeletal tissue engineering require the optimization of the degree of fiber alignment, a piezoelectric response, and good mechanical properties.^13,14,17,20,21^

It is well known that the various parameters of the electrospinning process such as the applied potential difference, the type of sample collector, the needle diameter, the needle to collector distance, the flow rate and the environmental conditions critically influence fiber properties.^11,22–24^ This brings various possibilities to steer and tailor electrospun fiber membranes for the required application.^11^ At the same time, such a large parameter window and their nonlinear dependance on the morphology and properties of the fibers makes electrospinning a challenging process for obtaining the desired properties.^25,26^ Therefore, the understanding of the multiscale structure–property relationship is essential to steer the properties of electrospun fibrous membranes accurately.

In the past, different techniques have been developed by applying external conditions such as mechanical, electrical, and magnetic forces to align fibers in a desired direction.^27–29^ One of the most straight forward ways to align fibers is to use a rotating drum collector.^28,30,31^ Several studies are reported to use a rotating drum collector to produce fiber membranes with varying degrees of orientation of the fibers within the membrane.^28,32,33^ However, producing electrospun fibers with desired properties (*e.g.*, degree of orientation, mechanical, surface properties, *etc.*) is primarily based on the trial-and-error method. In a previous study, we combined scanning electron microscopy (SEM), atomic force microscopy (AFM), small-angle X-ray scattering (SAXS) and wide-angle X-ray diffraction (WAXD) to decode the multiscale structure of non-aligned and aligned poly(vinylidene fluoride-*co*-hexafluoro propylene) (PVDF-HFP) electrospun fiber membranes at various speeds of the rotating drum.^30^ We reported that electrospun fibers possess a nanofibrillar structure with repeating lamellae with tie molecules in between and concluded that the moderate rotating speed of the rotating drum collector used in the study only influences the alignment of the fibers in the membranes but results in no change in the internal structure of the fibers.^30^ However, Kongkhlang *et al.* have reported that electrospinning can be used for controlling the morphology and crystal orientation in polyoxymethylene (POM) fibers by producing them at different rotating collector speeds.^34^ They reported that increasing the rotation speed of the rotating drum collector results in additional stretching on the fibers.^34^ This influences the molecular arrangement of the polymeric chains within the fibers.

Therefore, in this follow-up study, we hypothesized to produce electrospun fibers at a rotating drum collector speed 2.2 times higher (23.0 m s^−1^) than in our previous study,^30^ and the structural changes were compared with a lower rotating drum collector speed of 0.5 m s^−1^. We expected that the additional stretching due to an extremely high rotating speed of the drum collector will induce more piezoelectric β-phase of PVDF-HFP due to change in molecular confirmation as observed in the stress-induced deformed PVDF films and fibers.^35–37^ Furthermore, it has also been reported that the degree of alignment of the fibers in the membranes provides enhanced mechanical properties along the fiber alignment direction.^38,39^ Hence, a high rotating speed of the drum collector might result in the change of the mechanical properties and the deformation behavior of fiber membranes due to structural modifications. Consequently, in this study, we present the effect of the high-speed rotating drum on the μm–nm–Å scale structural changes and their correlation with the mechanical and piezoelectric properties of the PVDF-HFP membranes. SEM, SAXS and WAXS were used to quantify multiscale structural changes and β-phase content in both membranes. The mechanical properties and mechanical deformation behavior of the two membranes produced at a low and an extremely high rotating speed of the collector were investigated by *in situ* SAXS measurement using uniaxial tensile loading up to 100% strain.

## Materials and methods

### Electrospun fiber fabrication

PVDF-HFP polymer (*M*_w_ ∼400 000 g mol^−1^) and dimethylformamide (DMF) were purchased from Sigma Aldrich, Switzerland. PVDF-HFP pellets were dissolved in DMF resulting in a concentration of 35 w/v%. The mixture was stirred overnight on an orbital shaker to prepare a homogeneous polymer solution. A 3 ml syringe was used in the electrospinning setup with a blunt 21 G needle. A rotating drum collector of 10 cm diameter was used with 50 and 2200 rpm that correspond to 0.5 and 23.0 m s^−1^ linear speeds, respectively. The homogeneous polymer solution was emitted at a rate of 20 μl min^−1^ under a potential difference of 19 kV (+14/−5 kV) from a needle-to-collector distance of 25 cm. Electrospinning was performed at room temperature (∼23 °C) and with about 55% humidity.

### Scanning electron microscopy (SEM)

A scanning electron microscope (Hitachi S-4800, Hitachi-High Technologies, Illinois, USA) with an acceleration voltage of 5 kV and a current flow of 10 μA was used to image the fiber membranes. An 8 nm think layer of conductive gold–palladium was deposited on each sample before imaging. DiameterJ (a plugin for imageJ) was used to determine the diameter of the fibers.^40^ The average diameter of the fibers was determined by taking an average of 30 fibers from SEM micrographs.

### Small-angle X-rays scattering (SAXS) and wide-angle X-ray Scattering (WAXS)

SAXS and WAXS measurements were performed with a Bruker Nanostar instrument (Bruker AXS GmbH, Karlsruhe, Germany). This pinhole-collimation system was equipped with a micro-focused X-ray Cu source (wavelength Cu Kα = 1.5406 Å) and custom-built semitransparent beamstop providing a beam size of about 400 μm. A 2D MikroGap technology-based detector (VÅNTEC-2000) with 2048 × 2048 pixels with a pixel size of 68 × 68 μm^2^ was used. The SAXS experiment was performed at a sample-to-detector-distance (SDD) of 107 cm providing a resolvable scattering vector modulus (*q*) range from 0.07 to 2.20 nm^−1^. The WAXS experiment was performed at a SDD of 5 cm providing a resolvable *q*-range from 2 to 27 nm^−1^. The SDDs were calibrated with silver behenate and corundum for the 107 cm and 5 cm distances, respectively. Each sample and background (residual air) were measured for 2 hours for both SAXS and WAXS under moderate vacuum conditions (10^−2^ mbar pressure) to reduce air scattering. After the measurement, 1D profiles were extracted using DIFFRAC.EVA (Bruker AXS, version 4.1).

### *In situ* SAXS experiment: uniaxial tensile loading of the electrospun membranes

A tensile stage (Anton Paar, TS600) was used for *in situ* SAXS investigations of the structural deformation in electrospun fiber membranes under uniaxial tensile loading. A load cell capable of applying a tensile force in the range of 0 to 6 N was used in this experiment. A rectangular-shaped sample of 23.6 × 10 mm was mounted on the tensile stage. Afterward, the tensile stage was placed inside the Bruker Nanostar instrument (Bruker AXS GmbH, Karlsruhe, Germany) to perform *in situ* SAXS measurements. The tensile stage setup inside the sample chamber of the Nanostar instrument is shown in Fig. 4(a). The fiber membranes were stretched along the fiber alignment direction. The *in situ* SAXS experiment was performed at a SDD of 107 cm. The mounted electrospun membranes were continuously elongated with a waiting time of 30 minutes at 2.5, 5, 10, 25, 50, 75 and 100% elongation when SAXS frames were recorded. 1D-profiles were extracted using DIFFRAC.EVA (Bruker AXS, version 4.1).

### Orientation analysis of fibers inside the membranes

A new spatial cross-correlation method-based algorithm was further extended from our previous study to quantify the degree of alignment of fibers in the electrospun membranes from SEM images.^30^ Small dimensions (500 × 500 pixels) were taken from the SEM images at a random position and were used as reference images. The spatial cross-correlation coefficients (*R*) were calculated with cropped images of same dimensions by shifting pixel-by-pixel in each direction from −90° to +90° with 1° step size. Thereafter, the number of pixel values at half decay of the correlation coefficient (*N*_1/2_) was determined by exponential fitting for each rotating direction. To improve the statistics, the same procedure was repeated by randomly selecting 50 cropped boxes on the SEM images. The average of *N*_1/2_ was determined for each rotating direction; the *N*_1/2_*vs.* the orientation angle is obtained. Finally, the resulting curve was fitted with a Lorentzian function and the respective full width at half maximum (FWHM) was determined. The obtained FWHM was correlated with the misorientation width (MOW) of the fibers inside the membranes.

### Porod-correlation peak model fitting of SAXS data

A Porod-correlation peak model was used to fit the 1D-SAXS profiles after the background subtraction. The Porod-correlation peak model is given in eqn (1) and the details are discussed elsewhere:^30^1
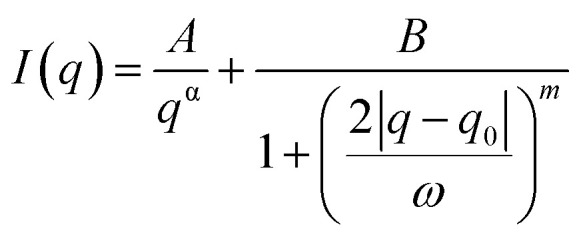
where *A* and *B* are the multiplying factors, *α* is the Porod exponent, *q*_0_ is the peak position, *ω* is the FWHM, and *m* is the fitting parameter for the peak shape correction. All fittings were performed in Matlab (R2019b).

The average lamellar spacing (*d*) was determined by using eqn (2).2
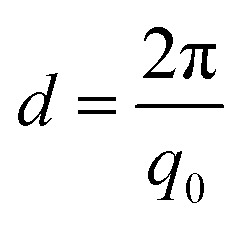


### Fitting of WAXS data to quantify the α/β-phase ratio

PVDF is co-polymerized with hexafluoropropylene (HFP), but due to the low crystallinity of the PVDF-HFP co-polymer, no crystal structure is available in the crystallographic database. However, PVDF and PVDF-HFP have been considered to possess similar crystal structures.^41,42^ Therefore, the monoclinic PVDF α (*P*2_1_/*c*) and β (*Cm*2*m*) phase crystallographic information provided by the Cambridge Crystallographic Data Center (CCDC) no. 1207416 and 1207418, respectively, was used for fitting.^43^ A total of 5 peaks (namely, amorphous, (100/020)_α_, (110)_α_, (100/200)_β_ and (021/111/120)_α_) were selected and fitted with a Gaussian function. Guan *et al.* also reported on using these main peaks to quantify the α and β-phases in the PVDF-HFP films.^42^ Due to the approximations used in our fitting, we aim only to highlight the relative changes between the patterns, in particular towards the α/β phase ratio, and do not report on absolute values.

## Results and discussion

### Microscale morphology of the fiber membranes

**Fig. 1:**
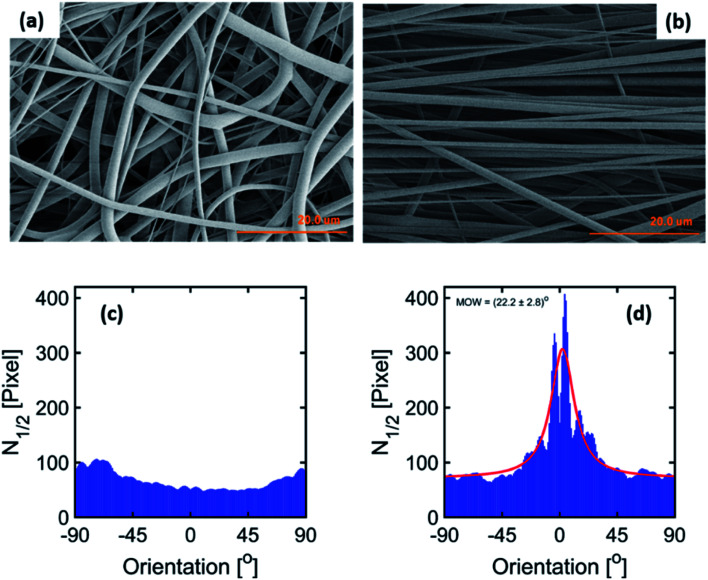
SEM micrographs of electrospun fiber membranes produced at (a) 0.5 m s^−1^ and (b) 23.0 m s^−1^. The orientation distribution plots of (c) 0.5 m s^−1^ and (d) 23.0 m s^−1^ membranes. The 0° orientation angle is the reference for the fiber alignment direction.

The parameters for the electrospinning of PVDF-HFP were optimized in our previous study.^30^ In this study, the rotating drum was set at a speed of 0.5 m s^−1^ and 23.0 m s^−1^ leading to the production of homogeneous non-beaded fiber membranes. The SEM micrographs of the fiber membranes are shown in Fig. 1(a) and (b) and macroscopic image in ESI Fig. S1.† The fibers obtained with the low rotating speed led to a randomly oriented fiber mesh, while with the high rotating speed the fibers were preferentially oriented. The fibers within the membrane electrospun at 0.5 m s^−1^ exhibited a diameter of 1.6 ± 0.1 μm, whereas the fibers electrospun at 23.0 m s^−1^ exhibited a diameter of 1.1 ± 0.1 μm. It means that the diameter of the fibers decreased at a sufficiently high rotation speed of the collector. It is, in general, proposed that the strong stretch force induced due to the high-speed rotating drum is responsible for the decrease in the diameter of fibers.^32,39^ Such a strong force might induce structural changes within the fibers as observed by Kongkhlang *et al.*^34^

### Determination of the degree of orientation using SEM micrographs of the membranes

The degree of orientation of the fibers inside the membranes was determined based upon our new spatial cross-correlation method-based algorithm which is shown in Fig. 1(c) and (d) for −90° to 90° orientation angles for the membranes produced at 0.5 m s^−1^ and 23.0 m s^−1^. The orientation angle of 0° is considered as the reference for the fiber alignment direction. The membrane produced at 0.5 m s^−1^ does not show any peak due to the random orientation of the fibers (Fig. 1(c)). A peak about 0° is observed for the membrane produced at 23.0 m s^−1^ rotation speed due to the orientation of the fibers (Fig. 1(d)); a MOW of (22.2 ± 2.8)° is obtained by peak fitting.

### Nanostructure modification observed by SAXS

**Fig. 2:**
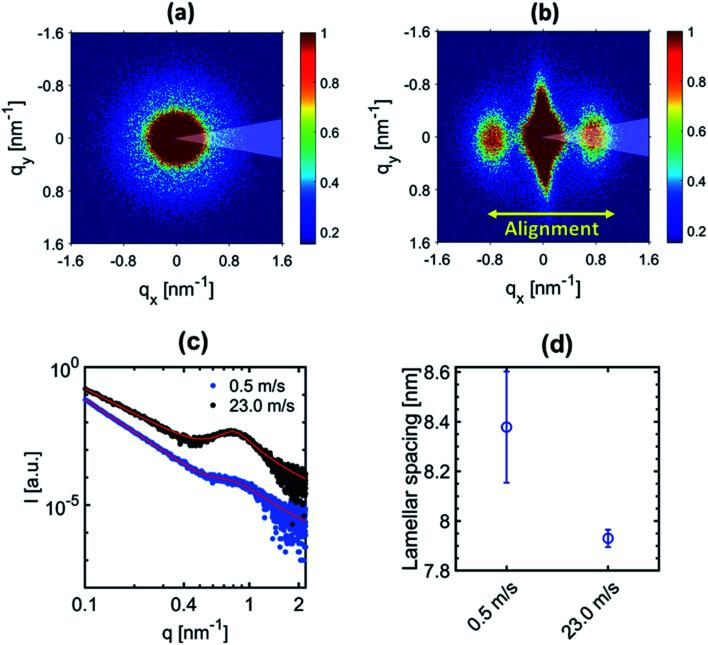
2D-SAXS profiles of PVDF-HFP electrospun fiber membranes produced at (a) 0.5 m s^−1^ and (b) 23.0 m s^−1^ rotating drum speeds. (c) 1D-radial profiles extracted along the fiber alignment direction (30° wedge azimuthal integration) and corresponding Porod-correlation peak model fittings (red continuous line). (d) Lamellar spacing for both samples.

The 2D-SAXS profiles of the electrospun fiber membranes at 0.5 m s^−1^ and 23.0 m s^−1^ are shown in Fig. 2(a) and (b) respectively. An isotropic scattering profile is observed for the sample produced at 0.5 m s^−1^ speed due to the random orientation of the fibers inside the membranes. However, the 2D-SAXS profile for the electrospun fiber membrane produced at 23.0 m s^−1^ speed depicts an anisotropic scattering profile related to the alignment of the fibers.

The peak observed in the 2D-SAXS profile along the fiber alignment direction reflect the periodicity of the crystalline (lamella) and amorphous domains within the fibers for the sample produced at 23.0 m s^−1^. Similarly, a halo is observed for the sample produced at 0.5 m s^−1^ due to the random distribution of fibers within the membranes. The center position of the peak or halo corresponds to the average distance, also known as lamellar spacing, between two adjacent lamella domains. In order to quantify the change in the lamellar spacing, 1D-radial profiles were extracted by performing azimuthal integration (30° wedge as indicated in Fig. 2(a) and (b)) on the 2D-SAXS patterns along the fiber alignment direction. The corresponding 1D extracted radial profile fitted with the Porod-correlation peak model given by eqn (1). The lamellar spacing is shown in Fig. 2(c) and (d). The peak position provides the lamellar spacing corresponding to the average distance between two adjacent lamella domains. The periodic repetition of lamella domains constitutes the nanofibrillar structure inside the electrospun fibers.^30^ A decrease in the lamellar spacing is observed for the fibers electrospun at an extremely high speed as shown in Fig. 2(d). However, no change in the lamellar spacing was observed in our previous study when fibers were produced at moderate rotating drum speeds up to 10.5 m s^−1^.^30^ This reflects that the polymeric chains of PVDF-HFP in the nanofibrillar structure of the fibers tend to be more aligned related to further mechanical stretching of the fibers collected at an extremely high rotating speed while negligible mechanical stretching occurs when the rotating speed was moderate. Such behavior of the rotating drum speed is due to the nonlinear dependence of the electrospinning parameter on the fiber morphology.^25,26^ Moreover, the decrease in lamellar spacing (increased polymer chain stretching) is the reason for the decrease in the diameter of the fibers.

### Quantification of the α/β-phase ratio using WAXS data

**Fig. 3:**
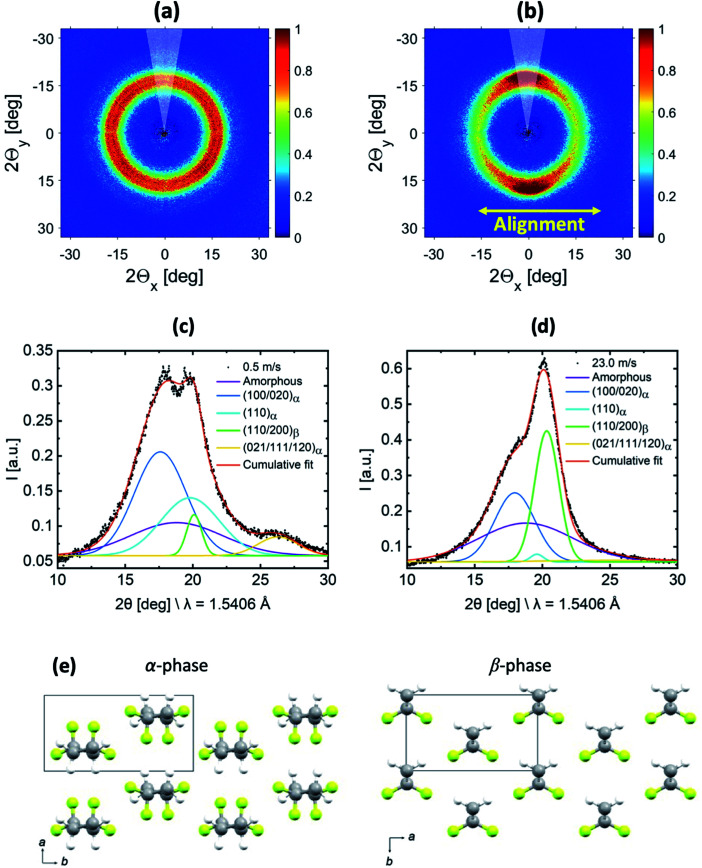
2D-WAXS profiles of the PVDF-HFP fiber membranes produced at (a) 0.5 m s^−1^ and (b) 23.0 m s^−1^ rotating drum collector speeds. 1D-radial profile extracted laterally to the fiber alignment (indicated by a 30° wedge in (a) and (b)) for the membranes produced at (c) 0.5 m s^−1^ and (d) 23.0 m s^−1^ with their corresponding fitting. (e) Crystal structures of the α and β-phases of PVDF from Hasegawa *et al.*,^44^ where the atom colours grey, yellow, and white represent C, F, and H, respectively.

WAXS measurements were performed to gain information about the relative amount of the α- and β-phases in the samples. The 2D-WAXS profiles of fiber membranes produced at 0.5 m s^−1^ and 23.0 m s^−1^ rotating drum collector speeds are shown in Fig. 3(a) and (b) respectively. Similar to SAXS, isotropic diffraction rings were observed for the sample produced at 0.5 m s^−1^ speed due to the random orientation of the fibers inside the membranes, while the anisotropic diffraction pattern for the fiber membrane produced at 23.0 m s^−1^ is related to the preferential alignment of the fibers.

**Fig. 4:**
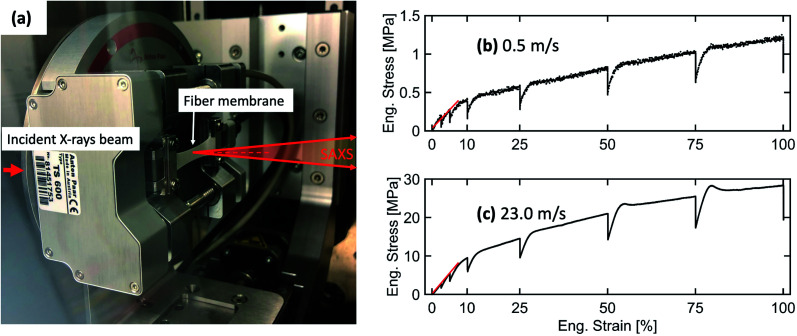
(a) Image of the mounted fiber membrane sample on the tensile stage placed in the sample chamber of the SAXS instrument. The engineering stress–strain curves with the linear fitting of the elastic region to determine Young's modulus for electrospun fiber membranes produced at (b) 0.5 m s^−1^ and (c) 23.0 m s^−1^ rotating drum speeds.

In order to characterize the evolution of the α- and β-phase fractions of the two membranes, the 1D-radial profiles were extracted laterally with respect to the fiber alignment direction from the 2D-WAXS data, which is shown in Fig. 3(c) and (d). Molecular drawings of α- and β-phases of PVDF including the respective unit cell information are shown in Fig. 3(e). A fitting was performed on the extracted 1D-radial profiles, which are shown in Fig. 3(c) and (d). The broad amorphous peak represents the amorphous phase centered at 18.8° for both samples. The peaks (100/020)_α_, (110)_α_ and (021/111/120)_α_ are at 17.8°, 19.8°, and 26.3° and represent the convoluted (100/020), (110) and (021/111/120) diffraction peaks of the α-phase, respectively. The peak (110/200)_β_ is centered at 20.3°, which represents the convoluted (110/200) diffraction peak of the β-phase. The peak positions correspond to the unit cell of α- and β-phases of PVDF as shown in ESI Fig. S2.† The relative fraction of α- and β-phases was determined for both samples from the ratios of the area under peaks. The area under peaks and related determined phases are summarized in supported Table S1.† We obtained an increase of about 8 times for the piezoelectric β-phase for the sample produced at 23.0 m s^−1^ compared to 0.5 m s^−1^ fiber membranes. The increase in the β-phase is because of the change in the molecular conformation (all-trans) induced due to the additional stretching of the polymer chains at an extremely high rotating speed.^35^

To support the increase of the β-phase in the fibers produced at 23.0 m s^−1^ compared to 0.5 m s^−1^, Fourier-transform infrared spectroscopy (FTIR) measurements were performed. An increase in the β-phase in the FTIR plot could be also observed for 23.0 m s^−1^ with respect to the intensity of the corresponding peaks in the recorded spectra (see ESI Fig. S3†). Even though FTIR spectra showed distinct β- and α-phase absorption bands, we relied on WAXS to determine the β-phase contents in the PVDF-HFP fibers due to the unavailability of the absolute absorption coefficients for PVDF-HFP required for calculation.

### Mechanical properties and deformation behavior of the membranes

#### *In situ* SAXS coupled with tensile loading

To further understand and explain the changes in mechanical properties and related electrospun membrane deformation behaviors with respect to the uniaxial tensile loading, *in situ* SAXS measurements were performed. The tensile loading was applied along the fiber alignment direction. The tensile stage setup with the loaded electrospun membrane inside the sample chamber of SAXS instrument is shown in Fig. 4(a). The engineering stress–strain curves are shown in Fig. 4(b) and (c) for fibers produced at 0.5 m s^−1^ and 23.0 m s^−1^, respectively. Some relaxation (decrease in stress under constant strain) in the engineering stress–strain curve is observed due to the waiting time, while the SAXS experiment was performed for 30 minutes. The engineering stress–strain curve represents elastic as well as plastic domains for both fiber membranes. Similar stress–strain behavior was also observed by Yano *et al.* for poly(vinyl alcohol) (PVA)^45^ and Camarena-maese *et al.* for polycaprolactone (PCL).^46^

The Young's modulus, yield stress, and yield strain were determined from the engineering stress–strain curve. The values are given in Table 1.

**Table 1:** Young's modulus, yield stress, and yield strain of the fiber membranes produced at 0.5 and 23.0 m s^−1^ rotating drum speeds

Mechanical properties	Fibers produced at 0.5 m s^−1^	Fibers produced at 23.0 m s^−1^
Yield stress [MPa]	0.4 ± 0.1	8.4 ± 0.1
Yield strain [%]	8.2 ± 0.2	8.2 ± 0.1
Young's modulus [MPa]	3.7 ± 0.2	68.0 ± 0.1

An increase in Young's modulus and yield stress was obtained for the fiber membrane produced at 23.0 m s^−1^ compared to the 0.5 m s^−1^ rotating drum collector speed because aligned fibers provide much higher resistance to the applied stress along their alignment direction.^34,46^ Interestingly, the yield strain remains the same for both membranes.

**Fig. 5:**
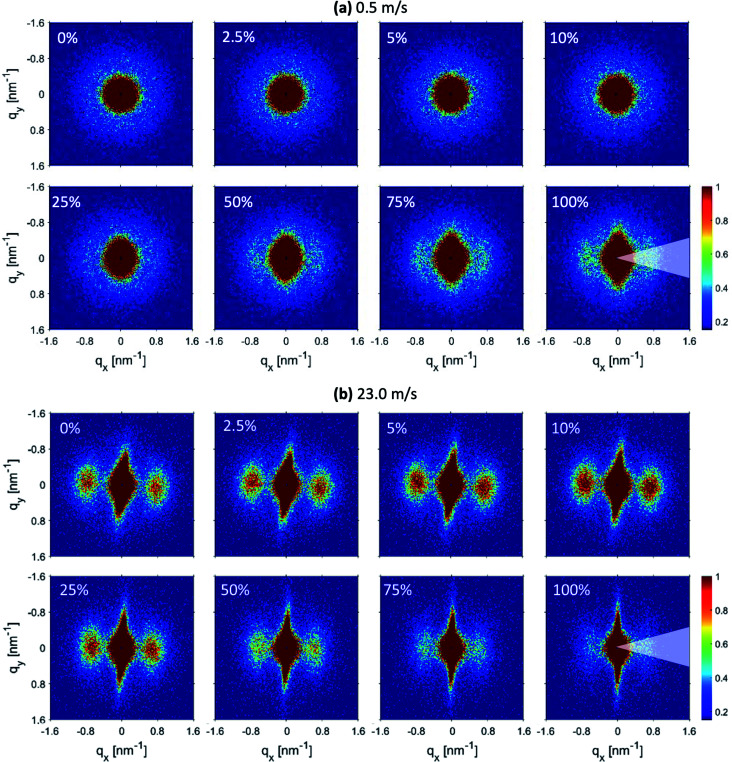
2D-SAXS profiles corresponding to increasing engineering strain for fiber membranes produced at (a) 0.5 m s^−1^ and (b) 23.0 m s^−1^ rotating drum speeds.

The 2D-SAXS profiles corresponding to an increasing percentage of engineering strain for electrospun membranes produced at 0.5 m s^−1^ and 23.0 m s^−1^ speeds are shown in Fig. 5(a) and (b) respectively. For the membrane produced at 0.5 m s^−1^ speed, the isotropic scattering gradually changes to anisotropic scattering upon increasing uniaxial tensile loading (Fig. 5(a)). It means that an increased number of fibers start to align along the loading direction.^45,46^ At 100% engineering strain, lamellar peaks start to appear more clearly in the 2D-SAXS profile. However, the membrane produced at 23.0 m s^−1^ speed was already aligned; therefore, when a uniaxial loading was applied, a streak-like signal started to increase while the peaks start to move towards the small *q* direction and finally disappear when 100% engineering strain is achieved (Fig. 5(b)). It could also be noticed that the intensity of the peaks increases up to 10% strain and then starts to decrease on further straining (Fig. 5(b)). This is direct evidence of a modification in the polymer chain arrangement on the nanoscale within the fibers.

**Fig. 6:**
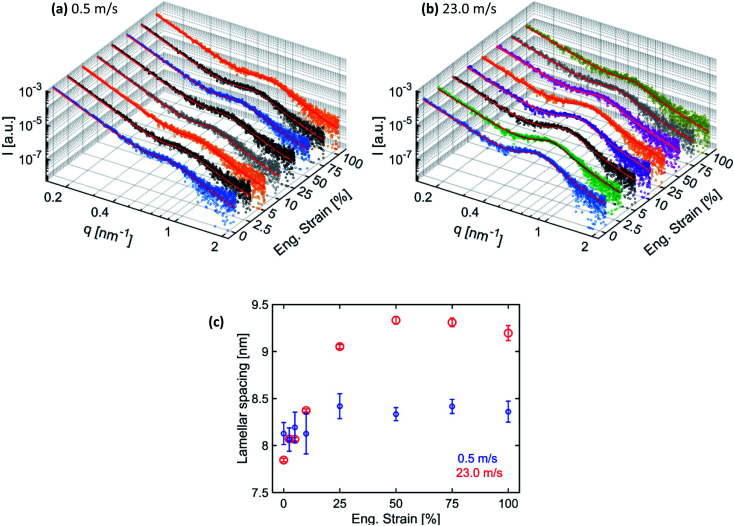
1D extracted radial profile (60° wedge azimuthal integration) from 2D-SAXS patterns in the direction of fiber alignment for membranes produced at (a) 0.5 m s^−1^ and (b) 23.0 m s^−1^ speeds with their corresponding fitting (continuous red line) and (c) determined lamellar spacing.

To quantify the changes in the lamellar spacing on increasing engineering strain, the 1D-radial profiles were extracted in the fiber alignment direction by performing 60° wedge azimuthal integrations. The extracted 1D-radial profiles were fitted with the same Porod-correlation peak model given by eqn (1). The 1D extracted SAXS radial profile together with the fitting is shown in Fig. 6(a) and (b) for the respective fiber membranes. The determined lamellar spacings are given in Fig. 6(c).

The lamellar spacing for the membranes produced at 0.5 m s^−1^ speed remains constant in the elastic region, while a slight increase with a plateau is observed in the plastic deformation domain. Fibers inside the membranes produced at 0.5 m s^−1^ speed under a uniaxial tensile loading up to 100% of engineering strain mainly get aligned with only little internal nanostructure modification in the plastic domain. However, the lamellar spacing for the membranes produced at 23.0 m s^−1^ speed increases linearly before reaching a plateau, followed by a decrease at 100% strain. In the case when most of the fibers were aligned, the uniaxial tensile loading along the alignment direction of the fibers is able to modify their nanostructure. The increase in the lamellar spacing is due to the elongation of the polymer chains in the amorphous region between the lamella domains.^47^ However, when the amorphous region with entangled polymer chains reaches the maximum elongation, then further increase in lamellar spacing is not possible and further stretching leads to the fracture of fibers. Consequently, lamellar spacing reaches a plateau and the intensity of the correlation peak also decreases.^46^ The decrease in lamellar spacing and the intensity of the correlation peak at 100% strain is probably due to the loss of electronic contrast between the lamella and amorphous domains as most of the polymer chains from amorphous domains get aligned due to stretching.

#### SEM micrographs of fiber membranes after 100% elongation in the relaxed state

**Fig. 7:**
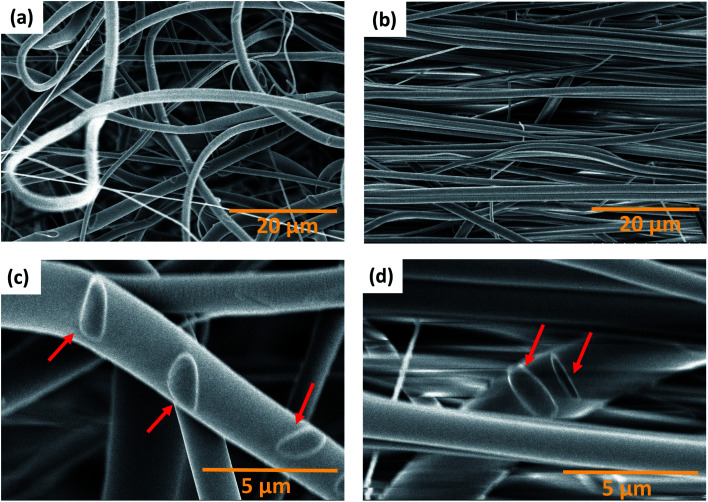
SEM micrographs of the PVDF-HFP membrane stretched after 100% elongation at (a) 0.5 m s^−1^ and (b) 23 m s^−1^: (c) and (d) zoomed-in fiber-to-fiber connections (oval shape) indicated by red arrows for 0.5 m s^−1^ and 23 m s^−1^, respectively.

SEM images were taken for both membranes after 100% uniaxial tensile loading in the relaxed state to observe the final stage and to compare with the SEM images of the pristine fiber membranes (Fig. 1(a) and (b)). The SEM images of the 100% strained membranes produced at 0.5 m s^−1^ and 23.0 m s^−1^ speeds are shown in Fig. 7(a) and (b) respectively, while Fig. 7(c) and (d) show the corresponding zoomed-in regions showing the fiber-to-fiber connection marks. The fiber membranes produced at 0.5 m s^−1^ speed still show entangled fiber networks even after 100% strain, while fibers produced at 23.0 m s^−1^ speed are highly aligned. Oval-shaped marks were observed for both fiber membranes (Fig. 7(c) and (d)) meaning that fiber-to-fiber connections could only be physical and no chemical bonds were formed during electrospinning.^48^ Therefore, when a tensile force was applied, the fibers were able to move apart leaving these oval-shaped marks behind. These marks also indicate poor evaporation of the DMF solvent during electrospinning.

Moreover, no fractured fibers were observed for both membranes up to 100% strain meaning that more force is required to achieve a fracture of the fibers. However, the fibers of the stretched membranes produced at 0.5 m s^−1^ have an average diameter of 1.5 ± 0.1 μm and the fibers of the stretched membrane produced at 23.0 m s^−1^ have an average diameter of 0.9 ± 0.1 μm. In comparison with the non-stretched fibers, the average diameter for the sample produced at 0.5 m s^−1^ remains unchanged, while a slight decrease was observed for the fibers produced at 23.0 m s^−1^. This can be explained by the fact that the fibers produced at 23.0 m s^−1^ were further stretched upon uniaxial tensile loading, which resulted in nanostructure modification as observed in the *in situ* SAXS experiment.

## Conclusion

This study shows that the fiber diameter, degree of alignment, β-phase content, and consequently the mechanical properties of electrospun PVDF-HFP fiber membranes can be steered by changing the speed of the rotating drum collector. The fibers produced at a higher rotating drum speed (23.0 m s^−1^) possess smaller diameters, a higher degree of alignment, and a change in polymer chain arrangement, which induces more piezoelectric β-phase content compared to fibers produced at a low speed (0.5 m s^−1^). The degree of orientation of fibers was quantified from SEM micrographs by the extension of our new image cross-correlation method-based algorithm. The increase in lamellar spacing was quantified by SAXS for fibers produced at 23.0 m s^−1^ and compared to 0.5 m s^−1^. An increase of about 8 times of the β-phase was obtained by fitting the WAXS data for membranes produced at 23.0 m s^−1^ compared to 0.5 m s^−1^. Moreover, the membranes electrospun at 0.5 m s^−1^ became more aligned after stretching but the diameter of the fibers did not change even at an elongation up to 100%. In addition, the membrane electrospun at 23.0 m s^−1^ demonstrated a change in the nanostructure of the fibers according to the *in situ* SAXS analysis. The diameter of the fibers decreased for this membrane at 100% elongation compared to the pristine fiber sample. This study contributes to the fundamental understanding of the multiscale structure–property relationship for producing electrospun fiber membranes with steered properties. The aligned fiber membranes investigated in this study with enhanced piezoelectric β-phase content and mechanical properties have potential applications in the field of neural or musculoskeletal tissue engineering.

## Conflicts of interest

There are no conflicts to declare.

## Supplementary Material

NA-004-D1NA00503K-s001
