# Supplementary material for: Understanding multiscale structure–property correlations in PVDF-HFP electrospun fiber membranes by SAXS and WAXS
Source: Nanoscale Adv. 2021 Nov 15;4(2):491–501. doi: 10.1039/d1na00503k (PMC8765355; doi:10.1039/d1na00503k)
Supplement: NA-004-D1NA00503K-s001 [file NA-004-D1NA00503K-s001.pdf]

Electronic Supplementary Material (ESI) for Nanoscale Advances.
This journal is © The Royal Society of Chemistry 2021

Understanding multiscale structure-property correlations in PVDF-HFP electrospun fiber membranes by SAXS and WAXS

Anjani K. Maurya[1, 2, 3], Eloïse Mias[1], Jean Schoeller[2,4], Ines E. Collings[1], René M. Rossi[2,4], Alex Dommann[1, 3], Antonia Neels*[1,5]

[1]*Empa, Swiss Federal Laboratories for Materials Science and Technology, Center for X-Ray Analytics, Lerchenfeldstrasse 5, 9014 St. Gallen, Switzerland*

[2]*Empa, Swiss Federal Laboratories for Materials Science and Technology, Laboratory for Biomimetic Membranes and Textiles, Lerchenfeldstrasse 5, 9014 St. Gallen, Switzerland*

[3]*ARTORG Center for Biomedical Engineering Research, University of Bern, Murtenstrasse 50, 3008 Bern, Switzerland*

[4]*ETH Zürich, Department of Health Science and Technology, 8092 Zürich, Switzerland*

[5]*Department of Chemistry, University of Fribourg, Avenue de l'Europe 20, 1700 Fribourg, Switzerland*

***Corresponding author**:
Antonia Neels, Email: antonia.neels@empa.ch
Empa, Swiss Federal Laboratories for Materials Science and Technology
Center for X-Ray Analytics,
Lerchenfeldstrasse 5, 9014
St. Gallen, Switzerland

S1. Macoscopic image of the fiber membrane

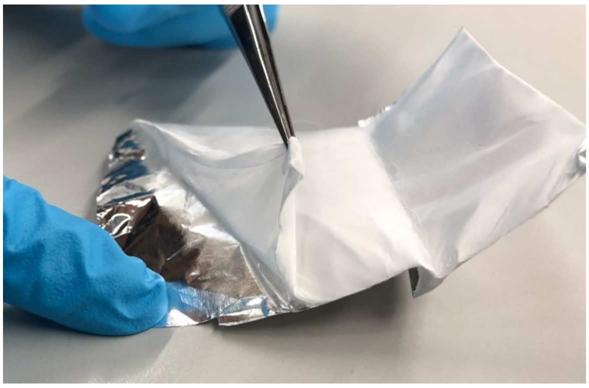

Figure S1: Image of the electrospun fibers membrane.

S2. Peak indexing based simulated α and β phases of PVDF

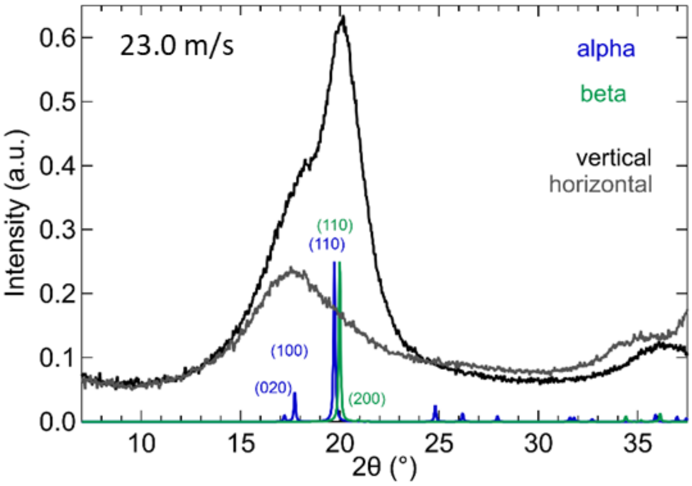

Figure S2. The diffraction of the fiber membrane prodiced at 23.0 m/s rotating speed integrated with a 30° wedge in the vertical (black line) and horizontal (grey line) directions. The simulated profiles of the alpha and beta phases are also shown with a preferred orientation down the (110) direction with a March-Dollase parameter of 0.5 using the Mercury software.

S3. The area under peaks determined by fitting of the WAXD data

Table S1: The determined area under peaks from the fitting of the WAXD data and calculated percentage of α- and β-phases.

**Area$_{sample}$**	**Amorphous**	**(100/020)$_\alpha$**	**(110)$_\alpha$**	**(110/200)$_\beta$**	**(021/111/120)$_\alpha$**
$A_{0.5m/s}$	0.40	0.71	0.45	0.08	0.10
$A_{23.0m/s}$	0.95	0.71	0.02	0.82	0.06

**Area (total α+β phases)**	**Area (β phase)**	**Area (total α phase)**	**%β**	**%α**
1.34	0.08	1.26	6	94
1.61	0.82	0.79	51	49

S4. Fourier-transform infrared spectroscopy (FTIR)

FTIR measurements were performed on a Bruker Vertex 80 FTIR spectrometer. For each sample, spectra were recorded at a range of 700 to 1500 cm^{-1}. The spectrometer was equipped with 3 integrated spheres, one for reflectance measurements in the solar range (0.3 to 2.5 μm). The IR detector was cooled with liquid nitrogen. FTIR provides information about the chemical composition of the materials. Therefore, it was used for quantifying the different phases of the PVDF-HFP.

The IR spectras were compared to the literature [1-3].

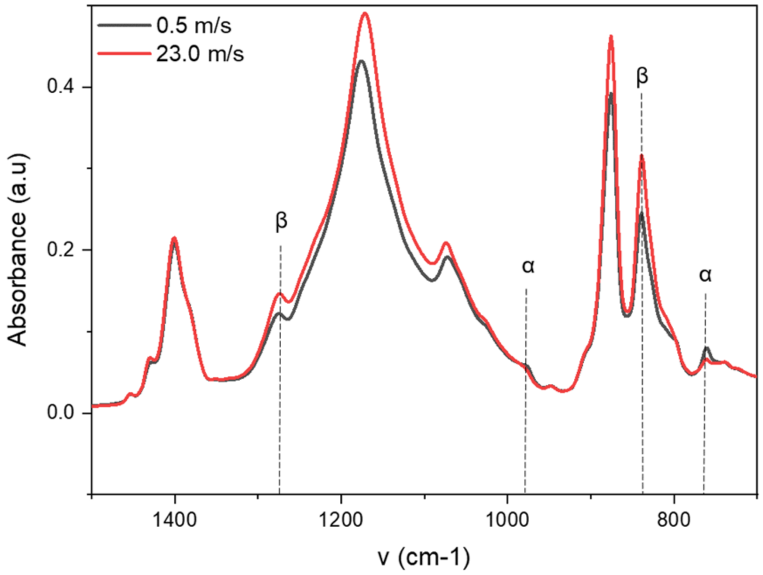

Figure S3: FTIR spectra of the electrospun fiber membrane produced at 0.5 m/s and 23.0 m/s rotating drum speed.

The β phase of PVDF-HFP is well-known to exhibit the highest electroactive properties [4] . According to figure S4, the PVDF-HFP electrospun fibers produced at 23.0 m/s contained more β phase than the electrospun membrane at 0.5 m/s speed. The membrane produced at 23.0 m/s exhibit high intensity at the wavenumbers of 1275 cm^{-1} and 840 cm^{-1}, which corresponds the main peaks for the β phase compared to membrane produced at 0.5 m/s speed. Moreover, at the wavelengths of 980 cm^{-1} and 760 cm^{-1}, which are the main peaks for *a*-phase, non aligned fibers produced at 0.5 m/s speed has higher interestity compeared to aligned fiber membranes produced at 23.0 m/s.

References

1. Gheibi, A., et al., *Electrical power generation from piezoelectric electrospun nanofibers membranes: electrospinning parameters optimization and effect of membranes thickness on output electrical voltage.* Journal of Polymer Research, 2014. **21**(11): p. 14.
2. Lanceros-Mendez, S., et al., *FTIR and DSC studies of mechanically deformed beta-PVDF films.* Journal of Macromolecular Science-Physics, 2001. **B40**(3-4): p. 517-527.
3. Salimi, A. and A.A. Yousefi, *FTIR studies of beta-phase crystal formation in stretched PVDF films.* Polymer Testing, 2003. **22**(6): p. 699-704.
4. Mokhtari, F., M. Latifi, and M. Shamshirsaz, *Electrospinning/electrospray of polyvinylidene fluoride (PVDF): piezoelectric nanofibers.* Journal of the Textile Institute, 2016. **107**(8): p. 1037-1055.
